# Alhagi; traditional and modern medicine effective against kidney stones

**Published:** 2015-08-26

**Authors:** Fatemeh Varshochi, Khairollah Asadollahi

**Affiliations:** ^1^Madani Heart Hospital, Lorestan University of Medical Sciences, Khorramabad, Iran; ^2^Department of Epidemiology, School of Medicine, Ilam University of Medical Sciences, Ilam, Iran

**Keywords:** Alhagi, Kidney stone, Iran

**Implication for health policy/practice/research/medical education:**

Urinary stones can cause kidney failure, urinary tract infections (UTIs) and severe abdominal pain, blood in urine and flanks pain. Alhagi has been seen in different regions of Iran, especially the north to the border of central deserts, and also grows in North Africa, Saudi Arabia, Syria, Iraq, Turkmenistan, Central Asia and other countries. Alhagi phytochemical such as flavonoids, flavone glycosides, Alhagidin, Alhagitin, proanthocyanidins, triterpenes, tannins, etc which it can be effective on urinary stones.

## Introduction

Urinary stones are of the most tragic causes of visiting hospitals. Although the cause of the infection and the incidence of kidney stones are not well known, while there are factors known involves in its development (1). Sex, water and age and its minerals, diet, climate and genetic are of the affecting factors (2). These patients are susceptible to renal failure, urinary tract infection (UTI) and usually suffer from severe abdominal and flanks pain (3). The common treatment for renal colic pain is using opioid compounds administration such as morphine and pethidine that have various side effects such as respiratory depression, hypotension and so on (4-6). Regarding less complications in kidney stones patients, in Iranian traditional medicines, it has been known numerous natural compounds which are effective to relieve kidney stones induced pain and have minimum side effects. Alhagi, *Alhagi pseudalhagi*, is a member of *Fabaceae* family are known as a painkiller in patients with kidney stones. Another name of Alhagi is manna grass which is plant of 20 to 120 cm height, with green to dark green prickly branches. Length of the thorns varies from 1 to 6 cm, and their angle is almost right. Leaves are oval-shaped with a width of 3 to 5 mm and a length of 10 to 15 mm, and the flowers which usually appear in 2 to 8 numbers in each thorns are red, purple and brown (7). This plant has been seen in different regions of Iran, especially the north to the border of central deserts, and also grows in North Africa, Saudi Arabia, Syria, Iraq, Turkmenistan, Central Asia and other countries (7,8). According to traditional medicine the nature of Alhagi is very hot and dry and has diuretic property and prevents kidney spasms, therefore, since ancient times, it has been used to alleviate kidney pain from kidney stones and urinary tract stones expulsion. In addition, it is efficient to attenuate UTI and renal colic (7). Results of experimental studies have shown 66% of patients treated Alhagi extract for 4 weeks expulsed urinary tract stones (9). The aqueous extract of Alhagi reduces calcium oxalate kidney stones (10). Alhagi phytochemical analysis have shown that the plant has bioactive and active pharmaceutical ingredients such as flavonoids, flavone glycosides, Alhagidin, Alhagitin, proanthocyanidins, triterpenes, tannins, etc. (11). It seems that the mentioned active ingredients are effective in reducing pain and kidney stones expulsion.

## Authors’ contribution

The authors contributed to the manuscript equally.

## Conflicts of interest

The authors declared no competing interests.

## Ethical considerations

Ethical issues (including plagiarism, data fabrication, and duplicate publication) have been completely observed by the authors.

## Funding/ Support

None.
